# Interocular induction of illusory size perception

**DOI:** 10.1186/1471-2202-12-27

**Published:** 2011-03-11

**Authors:** Chen Song, D Samuel Schwarzkopf, Geraint Rees

**Affiliations:** 1 UCL Institute of Cognitive Neuroscience, University College London, 17 Queen Square, London WC1N 3AR, UK; 2 Wellcome Trust Centre for Neuroimaging, University College London, 12 Queen Square, London WC1N 3BG, UK

## Abstract

**Background:**

The perceived size of objects not only depends on their physical size but also on the surroundings in which they appear. For example, an object surrounded by small items looks larger than a physically identical object surrounded by big items (Ebbinghaus illusion), and a physically identical but distant object looks larger than an object that appears closer in space (Ponzo illusion). Activity in human primary visual cortex (V1) reflects the perceived rather than the physical size of objects, indicating an involvement of V1 in illusory size perception. Here we investigate the role of eye-specific signals in two common size illusions in order to provide further information about the mechanisms underlying illusory size perception.

**Results:**

We devised stimuli so that an object and its spatial context associated with illusory size perception could be presented together to one eye or separately to two eyes. We found that the Ponzo illusion had an equivalent magnitude whether the objects and contexts were presented to the same or different eyes, indicating that it may be largely mediated by binocular neurons. In contrast, the Ebbinghaus illusion became much weaker when objects and their contexts were presented to different eyes, indicating important contributions to the illusion from monocular neurons early in the visual pathway.

**Conclusions:**

Our findings show that two well-known size illusions - the Ponzo illusion and the Ebbinghaus illusion - are mediated by different neuronal populations, and suggest that the underlying neural mechanisms associated with illusory size perception differ and can be dependent on monocular channels in the early visual pathway.

## Background

**Figure 1:**
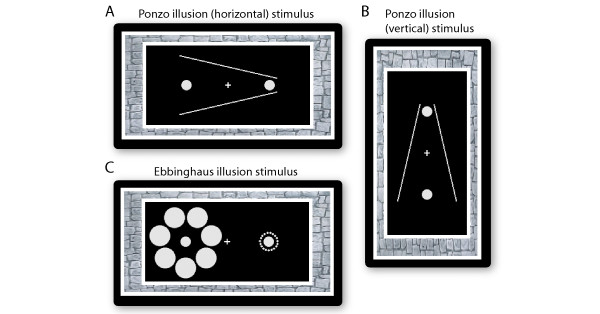
**Ponzo illusion stimulus and Ebbinghuas illusion stimulus**. In the Ponzo illusion (A, B), the two converging lines provided the depth impression that the two physically identical objects were located at different distances from the observer, and the distant object looks larger than the object that appears closer in space. In the Ebbinghaus illusion (C), the object surrounded by small items looks larger than a physically identical object surrounded by big items.

**Figure 2:**
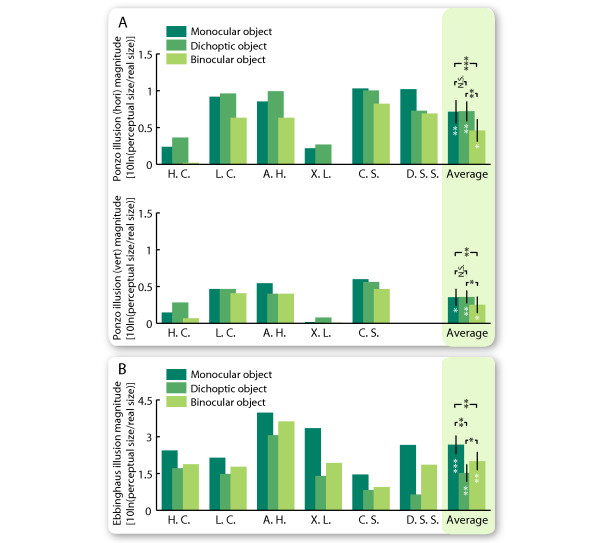
**Different interocular transfer patterns in the two illusions**. Both the Ponzo and the Ebbinghaus illusion persisted in all three presentation conditions. In the Ponzo illusion (A), the illusion magnitude is the same under monocular and dichoptic presentation, and lowest under binocular presentation. In the Ebbinghaus illusion (B), the illusion magnitude is highest under monocular presentation and lowest under dichoptic presentation. Error bars represent SEM (N = 6 or 5). * p < 0.05, ** p < 0.01, *** p < 0.001.

Accurate estimates of object size play an essential role in guiding our daily actions such as picking up a coffee cup and walking through a door. However, the perceived size of objects not only depends on their physical size but also on the surroundings in which they appear. Size illusions arise when the surroundings interact with our perception of the objects and lead to misjudgements of the size of the objects. For example, when two identical objects are placed in a context that suggests they are located at different distances from the observer (e.g., the Ponzo illusion, Figure 1A and 1B, [1]), the contextually more distant object appears to be larger than the closer one, as a result of the assumption incorporated by the visual system about the distance of each object from the observer. A similarly illusory perception of object size occurs in the Ebbinghaus illusion (Figure 1C, [2]), in which the size of items immediately surrounding an object determines whether the object is perceived to be larger or smaller than its physical size. A surround containing small items makes the object appear larger and vice versa. These size illusions not only trick our visual system but also distort our action, such that the grasping aperture will reflect the perceptual size instead of the physical size of an object [3].

Although the visual system has been intensively studied and a lot is known about the processing of simple object features such as orientation, luminance, colour, motion, and shape, surprisingly little is known about the neural processes underlying size perception. The spatial extent of neural activity in human primary visual cortex (V1) reflects the perceived size rather than the physical size of an object in a Ponzo-like illusion [4,5], suggesting a possible role for V1 in illusory size perception. The geniculostriate visual pathway is segregated into monocular pathways from the left and right eyes. The first stage at which information from the two eyes converges is V1, but it nonetheless contains populations of monocular neurons, which respond, with varying degree of exclusivity, to direct stimulation from only one of the eyes [6-8]. Therefore, it is unclear whether binocular or monocular neuronal populations of V1 are involved in the perception of the Ponzo illusion. Moreover, it remains unknown whether other forms of illusory size perception such as the Ebbinghaus illusion are mediated by the same or different neuronal mechanisms. The Ponzo and the Ebbinghaus illusions are induced by spatial contexts suggesting a role for different types of contextual information. While the contexts in the Ponzo illusion (Figure 1A and 1B) contain monocular depth clues that globally affect both objects, the contexts in the Ebbinghaus illusion (Figure 1C) are simple geometric patterns that locally affect the adjacent object but not the other object. Such discrepancy between the two illusions may be associated with the involvement of different neuronal populations.

Here we studied the extent to which monocular and binocular neurons in the human visual system were involved in two different size illusions: the Ponzo illusion and the Ebbinghaus illusion. We took advantage of the well-described functional organization of the visual system to infer the cortical stage at which the illusory size perception occurred. In visual cortices beyond V1 almost all neurons are binocular, whereas in subcortical visual areas (such as the lateral geniculate nucleus in the thalamus) and V1 a large proportion of neurons are monocular. Interocular transfer paradigms can reveal the degree of binocularity in illusory size perception and allow us to make inferences concerning the neuronal populations or neural stages involved [9,10]. If a spatial context in one eye exerts an influence on the perceived size of an object presented to the other eye, this suggests that the illusory size perception is mediated by binocular neurons at V1 or higher visual areas. Conversely, if the process is substantially reduced under dichoptic presentation (i.e., presenting the objects and their spatial contexts to different eyes), this implicates the involvement of monocular neurons early in the visual system such as at lateral geniculate nucleus (LGN) or V1.

We therefore devised stimuli in which an object and its spatial context associated with a size illusion could be presented together to one eye (monocular presentation) or separately to the two different eyes (dichoptic presentation). In separate experiments, we quantified the magnitude of illusory size perception induced by the two different (Ponzo vs. Ebbinghaus) illusions, and examined how the magnitude of the illusion was affected (if at all) by these interocular manipulations. We found that the Ponzo illusion showed equivalent magnitudes in dichoptic and monocular presentations, but the Ebbinghaus illusion in contrast was much weaker in dichoptic compared to monocular presentation.

## Results

We studied the two illusions under monocular, dichoptic, and binocular conditions: the spatial contexts were presented to only one eye, and the objects were presented to either the same eye (monocular), or the opposite eye (dichoptic), or to both eyes simultaneously (binocular). Contrasting the monocular and dichoptic conditions allowed us to evaluate the degree of interocular transfer in illusory size perception, whereas contrasting the binocular condition with the monocular and dichoptic conditions allowed us to infer the linearity vs. nonlinearity of the interocular transfer effect. For a better comparison of the Ebbinghaus and the Ponzo illusions, we matched the spatial configuration of the two illusion stimuli by using a horizontal configuration for the Ponzo illusion (Figure 1A, [11]). We also studied the vertical configuration of the Ponzo illusion (Figure 1B, [1]) to generalize the findings.

The Ponzo illusion persisted under all three conditions of stimulus presentation (Figure 2A; horizontal Ponzo illusion; monocular, t(5) = 4.6, p < 0.01; dichoptic, t(5) = 5.3, p < 0.01; binocular, t(5) = 3.0, p < 0.05; vertical Ponzo illusion; monocular, t(4) = 3.1, p < 0.05; dichoptic, t(4) = 4.3, p < 0.01; binocular, t(4) = 2.3, p < 0.05; right tailed t-test). However, the magnitude of the illusion changed over different conditions (horizontal Ponzo illusion, F(2,10) = 17.8, p < 0.001; vertical Ponzo illusion, F(2,8) = 5.6, p < 0.05; one-way repeated measures ANOVA). The illusion magnitude was the same when the objects and the contexts were presented to the same eye or to different eyes (monocular vs. dichoptic: horizontal Ponzo illusion, t(5) = 0.10, p = 0.93; vertical Ponzo illusion, t(4) = -0.07, p = 0.95; paired t-test), but decreased when the objects were presented to both eyes simultaneously (binocular vs. monocular: horizontal Ponzo illusion, t(5) = 13.2, p < 0.0001; vertical Ponzo illusion, t(4) = 6.4, p < 0.005; binocular vs. dichoptic: horizontal Ponzo illusion, t(5) = 4.9, p < 0.01; vertical Ponzo illusion, t(4) = 2.8, p < 0.05; paired t-test). To infer the (non)linearity of interocular transfer in illusory size perception, a regression model *B *=*β*_1 _* (*M *+ *D*) +*β*_2 _* M * *D *(in which *B*, *M*, *D *denotes the illusion magnitude under binocular, monocular, dichoptic conditions, respectively, and *M *+ *D*, M * *D *denotes the linear, nonlinear interactions, respectively) was used to fit the data. Results suggested that the interaction between the monocular and dichoptic conditions under the Ponzo illusion was nonlinear (*β*_1 _= -0.04 ± 0.28, *β*_2 _= 0.89 ± 0.63, 95% confidence interval; R^2 ^= 0.9649).

Similar to the Ponzo illusion, the Ebbinghaus illusion persisted regardless of the type of interocular manipulation (Figure 2B; monocular, t(5) = 7.4, p < 0.001; dichoptic, t(5) = 4.3, p < 0.01; binocular, t(5) = 5.6, p < 0.01; right tailed t-test), but changed in magnitude across different manipulations (F(2,10) = 16.0, p < 0.001, one-way repeated measures ANOVA). Interestingly, the change in the magnitude of the Ebbinghaus illusion showed a very different pattern from that of the Ponzo illusion. Presenting the objects and their spatial contexts to different eyes greatly weakened the Ebbinghaus illusion (dichoptic vs. monocular, t(5) = 4.3, p < 0.01; paired t-test). Further, when the objects were presented to both eyes simultaneously and the contexts to only one eye, the Ebbinghaus illusion had an intermediate magnitude (binocular vs. monocular, t(5) = 4.1, p < 0.01; binocular vs. dichoptic, t(5) = 2.9, p < 0.05; paired t-test), and the regression model suggested a linear interaction between the monocular and dichoptic conditions (*β*_1 _= 0.44 ± 0.15, *β*_2 _= 0.04 ± 0.12, 95% confidence interval; R^2 ^= 0.9333).

**Figure 3:**
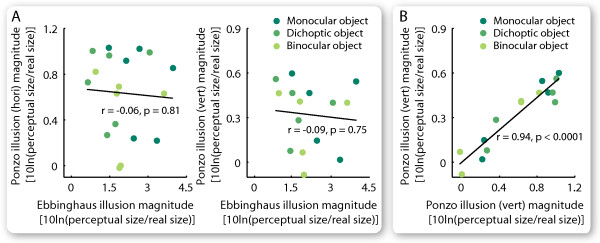
**Correlation in the illusion magnitude**. Each point represents the magnitudes of two illusions for one participant in one of the three conditions (monocular, dichoptic, binocular). Comparing the Ponzo illusion and the Ebbinghaus illusion (A), the illusion magnitudes were not correlated in strength for each separate condition (N = 6 or 5) or for all three conditions considered together (N = 18 or 15). Comparing the Ponzo illusion in the horizontal and the vertical design (B), the illusion magnitudes were correlated in strength for each separate condition (N = 5) and for all three conditions considered together (N = 15).

The different interocular transfer patterns in the two illusions suggested that they have different underlying mechanisms. Further intra-individual comparisons showed that while the magnitudes of the horizontal and the vertical Ponzo illusion are strongly correlated (Figure 3B; monocular, r = 0.97, p < 0.01, N = 5; dichoptic, r = 0.91, p = < 0.05, N = 5; binocular, r = 0.97, p = < 0.01, N = 5; all together, r = 0.94, p < 0.0001, N = 15), there is no correlation between the magnitudes of the Ebbinghaus and the Ponzo illusion (Figure 3A; Ebbinghaus - vertical Ponzo; monocular, r = -0.32, p = 0.54, N = 6; dichoptic, r = 0.13, p = 0.80, N = 6; binocular, r = -0.05, p = 0.92, N = 6; all together, r = -0.06, p = 0.82, N = 18; Ebbinghaus - horizontal Ponzo; monocular, r = -0.28, p = 0.65, N = 5; dichoptic, r = -0.09, p = 0.88, N = 5; binocular, r = 0.03, p = 0.96, N = 5; all together, r = -0.09, p = 0.75, N = 15).

## Discussion

Our study suggests that two well-known size illusions - the Ebbinghaus illusion and the Ponzo illusion - arise from different neuronal mechanisms. The Ponzo illusion showed complete interocular transfer; that is, the strength of the illusion was equivalent regardless of whether the inducing spatial context was presented to the same or different eye as the objects whose perceived size participants were asked to judge. Conversely, in a similar configuration there was significantly reduced interocular transfer for the Ebbinghaus illusion. The complete interocular transfer observed in the Ponzo illusion indicates that it is mediated by binocular neuronal populations where visual inputs from the two eyes are combined and the information about the eye of origin is lost. The induction of the Ebbinghaus illusion magnitude from monocular to dichoptic presentation indicates that it is in large part mediated by monocular neuronal populations. Thus, the neural mechanism underlying illusory size perception is likely to depend on the type of spatial contexts in which the objects appear.

In the early visual system, inputs from the left and right eye are clearly segregated, up until V1 where there exist neuronal populations with varying degrees of binocularity [6,7]. A dichoptically presented stimulus will drive responses in neurons exclusively responsive to the same eye (i.e., monocular neurons), as well as binocular neurons but with a decreasing degree depending on their ocular dominance. The weak interocular transfer that we observed for the Ebbinghaus illusion is therefore an indication that this illusion could be mediated by V1 or even earlier in the geniculostriate pathway (e.g., LGN) where the majority of the neurons are monocular. In contrast, the full interocular transfer that we observed with the Ponzo illusion provides strong evidence that this form of illusory size perception reflects activity in visual areas at least as high as V1.

The conclusion that the two illusions have different underlying neural mechanisms is further supported by the intra-individual comparisons that showed a lack of correlation between the magnitudes of each illusion across individuals. Moreover, when a regression model was used to fit individual illusion magnitudes under different interocular manipulations, this suggested a linear interocular interaction in the Ebbinghaus illusion but a nonlinear interaction in the Ponzo illusion. The Ebbinghaus illusion magnitude under binocular conditions reflected a linear combination of that under monocular and dichoptic conditions, whereas in the Ponzo illusion a nonlinear combination was present. Such differences are also indicative of a monocular component in the Ebbinghaus illusion and a binocular component in the Ponzo illusion. Since monocular signals from the two eyes are subjective to nonlinear neural processing in binocular summation [12,13], the nonlinearity in the Ponzo illusion suggests that the illusory size perception here takes place at or after the stage of binocular summation. At this stage, the illusion inducers, as inputs from a single eye, are weaker than the binocular inputs of objects, leading to the induction of illusion magnitude from monocular/dichoptic to binocular condition. In contrast, the linearity in the Ebbinghaus illusion indicates that the illusion takes effect separately at different monocular channels before binocular summation.

The discrepancy between the Ponzo and the Ebbinghaus illusion may be largely due to the difference in the spatial contexts that induce the illusory perception. While the contexts in the Ponzo illusion suggest three-dimensional depth/distance information, the contexts in the Ebbinghaus illusion are simple geometric forms/contours. Although the slant contexts in the Ponzo illusion are monocular rather than binocular depth clues, it is plausible that the processing of monocular depth clues also requires the engagement of binocular neurons [14] as does the processing of binocular disparity clues [15]. The simple geometric contexts in the Ebbinghaus illusion, on the other hand, are likely to be processed already in V1 where a large proportion of neurons are monocular [16]. Notably, the contexts in the Ponzo illusion globally affect both objects, whereas the contexts in the Ebbinghaus illusion locally affect the adjacent object but not the other object. As the receptive fields of neurons increase significantly in size along the visual pathway [17], the processing of global contexts are likely to be mediated by higher visual areas where the neuronal receptive fields are large, and that of local contexts may be mediated by neurons with small receptive fields in lower visual areas. Thus, the different cognitive natures of the two illusions may be the underlying cause of the different neuronal involvements.

Interestingly, similarly to our findings in the Ebbinghaus illusion, other contextual effects such as collinear facilitation [18] and the tilt illusion [19] also exhibit partial interocular transfer. In collinear facilitation, the threshold for detecting a low contrast Gabor patch is enhanced when it is flanked by two higher contrast patches sharing the same orientation [20,21]. In the tilt illusion, the perceived orientation of a central grating is biased by the orientation of the surrounding grating [22]. Both of these illusions are dependent on the local contexts of stimulus orientation, a feature known to be processed by V1 neurons [6,7]. The lateral intrinsic connections in V1 [23-25] which link neurons with similar receptive field properties [26] are hypothesized to modulate such contextual influences on visual perception [27]. It is conceivable that the Ebbinghaus illusion also depends on these lateral connections.

## Conclusions

Our study shows that monocular and binocular processing is involved in different ways in two phenomenally similar size illusions. While the Ponzo illusion was completely binocular and may largely involve higher visual areas, the Ebbinghaus illusion was primarily mediated by monocular pathways and therefore probably arises in primary visual cortex or subcortical visual areas. These findings suggest that the extent to which different neuronal populations are involved in illusory size perception is dependent on the spatial contexts in which the objects appear. This raises the possibility that different types of size illusions will affect our visually guided actions and interact with other visual pathways (e.g., color processing) in different ways. Such topics would be of interest for future study.

## Methods

### Participants

Six healthy, right-handed participants (4 females, 2 males, aged 22 to 32) with normal or corrected-to-normal vision participated in this study. All gave written informed consent, and the study was approved by the local ethics committee. Apart from two of the authors (CS and DSS), the other participants were naive to the aims of the experiments and received payment for participation. Five of the six participants took part in all three experiments, and one participant took part in experiment 1 and experiment 2.

### Apparatus

Visual stimuli were programmed in Matlab Psychtoolbox [28] and were presented on a calibrated CRT monitor (size 22", spatial resolution 1024 × 768 pixels, refresh rate 100 Hz). The experiments were conducted in a darkened room with the monitor providing the only significant source of light. The left-eye and the right-eye stimuli were presented on the left and the right half of the monitor, respectively, and the participants viewed the stimuli at a distance of 67 cm through a mirror stereoscope with a chin and forehead rest. The participants indicated their responses by pressing the assigned keys on a keyboard.

### Experiment 1

**Figure 4:**
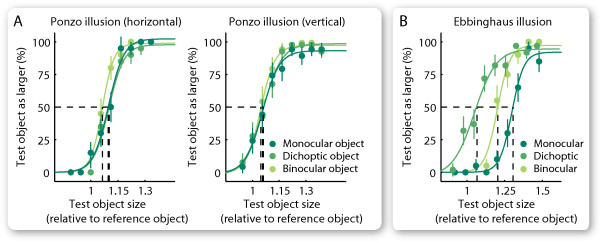
**Measurement of the illusion magnitude**. The size of one object (the reference object) was kept constant and the size of the other object (the test object) was varied. The percentage of the test object being judged as larger was plotted against the relative size of the test object (illustrative data from the Ponzo illusion in (A) and the Ebbinghaus illusion in (B)). The data were fitted with a logistic function, and the illusion magnitude was quantified according to the threshold point at which the test object was judged as larger for half of the trials (dashed lines). Error bars represent SEM (N = 20).

In experiment 1, we studied the perception of the Ebbinghaus illusion under monocular, dichoptic, and binocular presentation of objects. The illusion inducers (i.e., the spatial contexts that induce the illusion) were presented to one eye, and the objects were presented to either the same eye (monocular), or the opposite eye (dichoptic), or to both eyes simultaneously (binocular). We kept the size of one object (reference object) constant and varied the size of its counterpart (test object). The participants judged which of the two objects appeared larger. Psychometric curves were generated from participants' reports, and the magnitude of the illusion was quantified according to the threshold at which the test object was judged as larger for half of the trials (Figure 4).

#### Stimuli

The objects were two circles separated by a horizontal distance of 4 deg. The illusion inducers were seven big circles (1 deg in diameter) surrounding one object at a distance of 1.25 deg and nineteen small circles (0.1 deg in diameter) surrounding the other object at a distance of 0.4 deg (Figure 1C). The object surrounded by nineteen small circles was chosen as the reference object, and the object surrounded by seven big circles was chosen as the test object. Whether the small or big circles (and corresponding reference and test objects) appeared in left or right visual fields was randomized across trials. The diameter of the reference object was kept at 0.5 deg, and that of the test object was varied around 0.5 deg on a per-participant basis (see Procedures for details). These stimulus parameters were selected through preliminary experiments to maximize the magnitude of the illusion, while ensuring at the same time that there was adequate binocular fusion and no binocular suppression under dichoptic presentation.

To aid binocular convergence, a fixation cross (0.25 deg × 0.25 deg) was centered in the illusion stimuli, and a textured rectangle frame (inner edges: 8 deg × 4 deg; outer edges: 10 deg × 5 deg) was placed around the illusion stimuli. The circles and the fixation cross were at maximum luminance (white, 105 cd/m^2^), the background was at minimum luminance (black, 0.69 cd/m^2^), and the rectangle frame was inlaid with a stone texture for better binocular convergence.

#### Procedures

Before the start of the experiments, nonius lines/illusory stimuli were dichoptically presented on the monitor, and each participant adjusted the stereoscope as well as the stimulus location, till the left-eye and the right-eye stimuli were well fused. During the experiments, the participants were instructed to press a special key if the left-eye and the right-eye stimuli were not well fused (e.g., the objects and the illusion inducers were misaligned), and the same trial was then repeated. For all six participants, less than 5% of the trials were reported not well fused.

The experiment consisted of two parts. In the first part, the participants adjusted the size of the test object to perceptually match the size of the reference object. Four adjustments were made for each presentation condition (monocular, dichoptic, binocular). In two adjustments, the test object surrounded by seven big circles was presented to the left of the fixation cross, and the reference object surrounded by nineteen small circles was presented to the right of the fixation cross. In the other two adjustments, the spatial configurations of the two objects (i.e., left or right of the fixation cross) were reversed.

After the first part, nine size values, which spread around the average result of the twelve adjustments (three presentation conditions times four adjustments), were chosen. The nine values were -0.1 deg, -0.075 deg, -0.05 deg, -0.025 deg, 0 deg, 0.025 deg, 0.05 deg, 0.075 deg, 0.1 deg away from the average adjustment value, and they were used as the size of the test object for the second part. In the second part, after 500ms presentation of the illusion stimuli, participants judged which of the two objects (the one to the left or the one to the right of the fixation cross) was the larger one. Twenty trials were tested for each combination of presentation condition and object size (three presentation conditions times nine object sizes resulting in a total of twenty-seven combinations). The test object was presented to the left of the fixation cross for ten trials and to the right for the other ten. The sequence of the trials was randomized and counterbalanced for the presentation condition (monocular, dichoptic, binocular), the test object size, and the test object location (i.e., to the left or right of fixation). After each trial, a high-contrast, dynamical, coloured noise stimulus was presented for 1000ms to prevent any potential interference between trials.

### Experiment 2

In experiment 2, we studied the Ponzo illusion under monocular, dichoptic, and binocular presentation of objects. For a better comparison of the results from experiment 1 and experiment 2, we matched the spatial configuration of the Ponzo illusion stimulus to that of the Ebbinghaus illusion stimulus. The two objects in the Ponzo illusion were aligned horizontally [11] instead of vertically [1], and similarly, the illusion inducers were two lines converged horizontally instead of vertically (Figure 1A). The two converging lines (length: 4.8 deg; width: 0.06 deg; converged at 25 deg) provided the depth impression that the two objects were located at different distances from the observer. Again, whether the lines converged from left to right or right to left (and the corresponding position of test and reference object) was randomized from trial to trial. The circle that should be perceived as farther away from the observer was chosen as the reference object, and the circle at the front side was chosen as the test object. We also matched the spatial distance between the objects and their surrounds in the two illusions; the converging lines in the Ponzo illusion surrounded the test object at a spatial distance of 1.25 deg and the reference object at 0.4 deg. Except for the above-mentioned changes in the stimulus parameters, experiment 2 was identical to experiment 1.

### Experiment 3

In experiment 3, we studied the Ponzo illusion with conventional vertical inducers (Figure 1B) [1] under monocular, dichoptic, and binocular presentation of objects. The parameters and procedures of experiment 3 were identical to those of experiment 2, except that the objects and the illusion inducers were in the vertical instead of the horizontal configuration.

### Data Analyses

Data from the second part of each experiment were used to calculate the illusion magnitude. The percentage of the trials in which the test object was judged as larger was plotted against the relative size of the test object (i.e., the size of the test object divided by that of the reference object). The Matlab curve fitting toolbox was used to fit the data with a logistic function (see Table 1 for the goodness of the fit). We quantified the illusion magnitude according to the threshold at which the test object was judged as larger for half of the trials. The illusion magnitude was calculated as 10ln(x), in which x denotes the relative size of the test object at the threshold point. Because the illusion strength is a ratio, the logarithmic transformation corrects for non-linearities and the zero value in the illusion magnitude corresponds to the case when the relative size of the test object was 1 at the threshold point. To test whether the illusion existed (i.e., whether the illusion magnitude is higher than zero), a right-tailed t-test was chosen. In all but one case (binocular condition for vertical Ponzo illusion) the effect would also have been significant with two-tailed tests.

**Table 1:** R^2 ^of data fitting with logistic function

	Exp2. Horizontal Ponzo			Exp3. Vertical Ponzo			Exp1. Ebbinghaus		
	**Mono**.	**Dich**.	**Bino**.	**Mono**.	**Dich**.	**Bino**.	**Mono**.	**Dich**.	**Bino**.

H.C.	0.9993	0.9980	0.9942	0.9881	0.9637	0.9705	0.9469	0.9901	0.9855

L.C.	0.9853	0.9848	0.9972	0.9616	0.9300	0.9943	0.9906	0.9945	0.9812

A.H.	0.9889	0.9840	0.9826	0.9988	0.9997	0.9987	0.9852	0.9539	0.9593

X.L.	0.9815	0.9704	0.9467	0.9983	0.9923	0.9826	0.9949	0.9778	0.9334

C.S.	0.9962	0.9962	0.9965	0.9900	0.9865	0.9966	0.9987	0.9952	0.9877

D.S.S.	0.9968	0.9933	0.9948				0.9879	0.9678	0.9860

R^2 ^value of logistic fitting in illusion magnitude measurement is listed for each participant in each condition (monocular, dichoptic, binocular) of each illusion (Ponzo illusion in horizontal design, Ponzo illusion in vertical design, Ebbinghaus illusion). The R^2 ^value reflects the goodness of the fit and justifies the use of logistic fitting to quantify the illusion magnitude.

## Authors' contributions

Conceived and designed the experiments: CS, DSS, and GR. Performed the experiments and analyzed the data: CS. Wrote the paper: CS, DSS, and GR. All authors read and approved the final manuscript.
